# A Series of Twenty Plates, Illustrating the Causes of Displacement in the Various Fractures of the Extremities

**Published:** 1835-07-01

**Authors:** 


*A Series of Twenty Plates, illustrating the Causes of Displacement in the various Fractures of the Extremities.* By G. W. Hind, M.R.C.S., formerly House-Surgeon to the Middlesex Hospital; late Curator to the Museum of Anatomy in the University of London.—London, 1835. Folio, pp. 48.

A scientific book may excel in two ways: it may announce original investigations,—and this is the first and highest species of merit: the second, and humbler, path of excellence consists in the more lucid and intelligible representation of the discoveries of others. The present work pretends only to belong to the second class, but deserves to rank high in it, for the beauty and clearness of the plates, and the skill that has been shewn by the author in the selection of his subjects. They comprehend fractures of the clavicle, the coracoid and acromion processes, and neck of the scapula; of the humerus at its neck, above and below the insertion of the deltoid, and above the condyles; of the coronoid and olecranon processes and shaft of the ulna; of the neck and shaft of the radius, with fractures of both radius and ulna in the middle and at the lower extremities; of the fingers; of the femur, at its trochanter major, at its neck, below the trochanter minor, at the shaft, and above the condyles; of the patella; of the tibia and fibula, with Pott's fracture of the one, and of the malleolus internus of the other. Each of these subjects is treated, first with reference to its causes, then the appearances it presents, and afterwards the causes of the displacement, concluding with a summary of the treatment to be pursued.

We can give our readers no idea of the faithfulness and remarkable beauty of the representations; but the following extract will show the author's explanatory powers, and his accurate knowledge of his subject.

*Fracture of the Neck of the Radius.*

" *Causes of Fracture.* This fracture is of very rare occurrence, and has been known to have taken place from the immediate application of violence to the part by means of machinery, the arm being at the same time fixed by means of the hand grasping a bar: in this situation the radius is prominent on the outer part of the arm, and is there exposed in the greatest degree to injury applied from without.

" *Appearances.* In the fracture of the neck of this bone, much difficulty will be found in detecting its immediate situation. The position of the arm at once indicates fracture of the radius; for it is invariably the case, in fracture of this bone, that the forearm is in a state of pronation and retains this position. The motions of the forearm, viz. that of pronation and supination, are entirely destroyed, but the extent of pronation is not carried beyond what is natural; (as is the case in all fractures of the radius, this only excepted:) little deformity takes place in fracture at this situation, from the circumstance of the parts being so thickly clothed and surrounded with muscles; the only deviation that is observed is a slight fulness on the anterior and upper part of the forearm, attended with inability to rotate it.

" *Causes of Displacement.* The upper portion of the bone will be found drawn slightly outwards and downwards, but not to any considerable extent. It will be remembered, that the radius above the fracture, or even perhaps at the immediate situation of the fracture, is surrounded by the annular ligament, which prevents its displacement beyond a certain limit, unless laceration has taken place. The action of the supinator radii brevis is the sole cause of the displacement of this portion of the bone: it arises from the outer condyle of the humerus, and is inserted into the anterior part of the head and upper extremity of the radius; its action is to rotate the radius outwards; the few fibres attached above the fracture roll the head of the bone outwards, and likewise give the broken surface the same direction. The lower portion is drawn forwards, upwards, and inwards. The biceps muscle, which is inserted into the tubercle of the radius, and consequently into the upper extremity of the lower portion, is, in the natural state of the parts, a flexor of the forearm through the medium of the radius: independently of this action, it likewise, under certain circumstances, becomes a supinator: the latter of these actions, when fracture has taken place at this point, is entirely lost, in consequence of the separation of the shaft from the upper fixed point upon which it moves; the lower portion of the bone is then drawn forwards and slightly upwards by the action of the biceps, producing the fulness on the anterior part. But it must be remembered, that the oblique ligament of the radius is now on the stretch, as well as the fibres of the supinator radii brevis, which are in action: these counteract in some degree the effect of the biceps muscle. It will likewise be observed that the lower portion is drawn inwards: this displacement depends upon the action of the pronator teres, which arises from the inner condyle of the humerus, and is inserted into the middle and outer part of the radius: its action is to draw the radius over the ulna, (to pronate the arm,) and it is consequently the cause of the displacement; for the bone, having now no fixed point above upon which it can move, is made by its contraction to take the direction inwards. These facts account at once for the difficulty which exists in distinguishing the crepitus, for the broken surfaces look in opposite directions; the upper being directed downwards and outwards, while the lower is directed inwards and upwards.

"*Treatment.* In order to distinguish the crepitus, the upper part of the forearm should be grasped by the hand; the thumb pressing at the upper and outer part below the joint, and pushing inwards the head of the radius, while the fingers at the same time draw the lower portion outwards: the arm being new rotated, the crepitus is discovered.

"The treatment in this fracture consists in flexing the fore-arm, and placing it in a state between pronation and supination, in which situation the lower portion of the bone is held in a state of perfect rest; for in this position the biceps, supinator brevis, and pronator teres, are thrown into a state of relaxation, together with the mass of flexor and extensor muscles. A roller should be applied from the hand to the elbow; previously to which a soft compress should be placed external to the head of the radius, and the bandage passed firmly around, securing it in its situation; by means of which the head of the bone is forced inwards and retained in its natural situation. A broad splint applied both to the palmar and dorsal surfaces of the fore-arm retain the bones in their natural situations." (P. 19.)

